# Extracellular Vesicles and Ebola Virus: A New Mechanism of Immune Evasion

**DOI:** 10.3390/v11050410

**Published:** 2019-05-02

**Authors:** Michelle L. Pleet, Catherine DeMarino, Spencer W. Stonier, John M. Dye, Steven Jacobson, M. Javad Aman, Fatah Kashanchi

**Affiliations:** 1 Laboratory of Molecular Virology, School of Systems Biology, George Mason University, Manassas, VA 20110, USA; mpleet@gmu.edu (M.L.P.); cdemarin@gmu.edu (C.D.); 2 Emergent BioSolutions, Gaithersburg, MD 20879, USA; stoniers@ebsi.com; 3 Virology Division, U.S. Army Medical Research Institute of Infectious Diseases, Fort Detrick, Frederick, MD 21702, USA; john.m.dye1.civ@mail.mil; 4 Viral Immunology Section, Neuroimmunology Branch, National Institute for Neurological Disease and Stroke, National Institutes of Health, Bethesda, MD, 20892, USA; jacobsons@ninds.nih.gov; 5 Integrated BioTherapeutics, Inc., Gaithersburg, MD 20850, USA; jaman@integratedbiotherapeutics.com

**Keywords:** Ebola virus, exosome, extracellular vesicles, VP40, NP, GP, cytokine

## Abstract

Ebola virus (EBOV) disease can result in a range of symptoms anywhere from virtually asymptomatic to severe hemorrhagic fever during acute infection. Additionally, spans of asymptomatic persistence in recovering survivors is possible, during which transmission of the virus may occur. In acute infection, substantial cytokine storm and bystander lymphocyte apoptosis take place, resulting in uncontrolled, systemic inflammation in affected individuals. Recently, studies have demonstrated the presence of EBOV proteins VP40, glycoprotein (GP), and nucleoprotein (NP) packaged into extracellular vesicles (EVs) during infection. EVs containing EBOV proteins have been shown to induce apoptosis in recipient immune cells, as well as contain pro-inflammatory cytokines. In this manuscript, we review the current field of knowledge on EBOV EVs including the mechanisms of their biogenesis, their cargo and their effects in recipient cells. Furthermore, we discuss some of the effects that may be induced by EBOV EVs that have not yet been characterized and highlight the remaining questions and future directions.

## 1. Introduction

### 1.1. Ebola Virus

Ebola virus (EBOV) is an enveloped, single-stranded RNA (−) virus known to cause severe hemorrhagic fever in both humans and non-human primates (NHPs). Historically associated with sporadic outbreaks in Central Africa, EBOV more recently gained notoriety during the largest outbreak to date in West Africa (focused in Sierra Leone, Guinea, and Liberia), where the virus was responsible for >11,000 cases and >28,000 deaths [1]. At the conclusion of this outbreak in 2016, EBOV once again faded from the public spotlight; however, outbreaks have continued to occur, particularly in the Democratic Republic of the Congo (DRC), where the virus is now considered endemic [2,3].

The EBOV genome is ~19 kb long and encodes seven open reading frames which translate to seven structural proteins. These structural proteins are the viral glycoprotein (GP_1,2_), nucleoprotein (NP), RNA-dependent RNA polymerase (L), and viral proteins 24 (VP24; minor matrix protein), VP30, VP35 and the major matrix protein VP40. Two additional non-structural proteins are also produced by transcriptional processing of the GP mRNA—a soluble GP (sGP) and a small soluble GP (ssGP) [4,5,6,7]. It is estimated that only approximately 20%–25% of GP products are the membrane-anchored GP_1,2_ form, while the remaining 75%–80% are of the secreted type, with ~70% sGP and ~5% ssGP [7,8,9,10]. During early infection, EBOV targets mainly monocytes/macrophages and dendritic cells (DCs). It is thought that these migrating cells aid in systemic dissemination of the virus throughout the host, especially into secondary lymphoid organs and the liver. Intense replication of the virus then takes place in other host cell types, including endothelial cells, epithelial cells, fibroblasts and hepatocytes [11,12,13,14]. Fatal filovirus infections often involve severely dysregulated innate and adaptive immune responses, defective coagulation, and are typified by systemic cytokine storm and multi-organ failure. Immune system damage includes type-I and type-II interferon (IFN) antagonism by VP35 and VP24, respectively, NK and T-cell depletion known as bystander lymphocyte apoptosis, and impaired DC maturation [15]. Severe presentations of disease are accompanied by viral replication within and necrosis of the spleen, liver, kidneys, gonads, gastrointestinal tract and endocardium [13,16].

In those who are fortunate enough to survive acute Ebola virus disease (EVD), the virus becomes undetectable in the blood, depicting a typical phenotype of viral clearance. However, either entire virus or viral components may remain detectable in patients relatively long after recovery, particularly in immune-privileged sites [17,18,19,20,21,22,23,24]. Notably, recent studies have shown that over 1 in 4 male survivors from the 2016 outbreak contained virus within their semen up to 7–9 months past the disappearance of EVD symptoms. Furthermore, virus has still been detectable up to 16–18 months later [17]. This type of “clinical latency” or persistent infection has significance for public health in the following ways: (1) viral reservoirs within immune privileged sites have implications for reemergence and persistence of the virus within the infected individual, potentially allowing for resurgence of infection, morbidity, and mortality [25]; (2) these reservoirs may allow for the transmission of the virus to additional individuals long after initial infection, hindering efforts to control the spread of outbreaks by public health infrastructures [25]; and (3) significant political, socio-economic, and social stigma considerations must be taken into account for policies to support the normal life and travel of EVD survivors [26,27,28,29]. Spread of the virus by this route is of particular concern, as several cases of sexual transmission from male to female have already been documented. One of the first such cases involved transmission from a man 6 months past his recovery to a woman in Liberia [30,31]. To date, three additional “flare-ups” of EBOV by sexual contact have been determined, together accounting for approximately half of the eight mini-outbreaks caused by persistently infected survivors documented since 2016 [32,33,34,35]. The most concerning of these cases occurred over 480 days (nearly 17 months) past the initial recovery of the patient [35]. To further complicate the matter, there have been multiple reports addressing potential asymptomatic or undiagnosed portions of the population. Previous studies have put the number of seropositive but undiagnosed/asymptomatic individuals anywhere between 0%–47% [36,37,38,39,40,41,42,43,44]. This of course implies that such individuals may represent an invisible source of viral transmission and future outbreaks. Exemplifying this is a case involving an asymptomatic mother who likely transmitted EBOV to her 9-month-old infant (as determined by sequencing and phylogenetic analysis) through breast milk, ultimately resulting in the fatality of the child [19]. Interestingly, the asymptomatic father’s semen was also EBOV-positive, but was distantly related to the strains found in the mother and child. Currently, the mechanism of this viral persistence is not well characterized, although new studies have hinted as several possibilities, which will be discussed later.

### 1.2. Extracellular Vesicles

Extracellular vesicles (EVs) are small, membrane-bound vesicles that are released from numerous cell types and are involved in cell-to-cell communication. The intercellular communication is mediated by EV cargo, consisting of nucleic acids and proteins, which can be transferred between cells to elicit a phenotypic change in the recipient cell [45,46,47]. EVs are heterogeneous; as such, the current literature separates EVs into several different categories based mainly on differences in size and cellular origin, including subtypes such as exosomes and microvesicles [48]. Microvesicles bud directly from the plasma membrane and are generally larger than exosomes with diameters ranging from 100 to 1000 nm. Exosomes, which are formed from the fusion of multivesicular bodies (MVBs) with the plasma membrane, are the smallest type of EV, and range approximately from 30 to 150 nm in diameter [49,50]. Exosomes are formed from the invagination of the endosomal membrane, leading to the formation of intraluminal vesicles (ILVs) within the endosome, which is subsequently termed an MVB. During biogenesis, numerous soluble factors such as cytosolic proteins and nucleic acids are captured and incorporated into these ILVs [45,46,49,50]. These components can be preferentially packaged through interactions with the endosomal sorting complexes required for transport (ESCRT) machinery. The ESCRT proteins form a series of cytosolic protein complexes described as the ESCRT-0, -I, -II, -III, and Vps4-Vta1 complexes [51,52]. These complexes are serially recruited to the endosome to form a pathway that recognizes, recruits and passes ubiquitinated cargo intended for disposal (i.e., through exocytosis or by degradation via lysosomal fusion) into nascent ILVs. Release of this preferentially packaged cargo within exosomes can then take place upon fusion of the MVB with the plasma membrane. Although classical formation of ILVs and exosomes has been characterized to take place mainly via ESCRT-mediated packaging, alternative mechanisms of exosomal biogenesis have been demonstrated. This was initially observed through the generation of ILVs in cell lines despite the functional knockout of all four ESCRT complexes [53]. These proposed ESCRT-independent routes include CD63-mediated interactions [54,55], and by means of lipids such as neutral sphingomyelinase (nSMase; ceramide-dependent exosome formation) and phospholipase D2 (PLD2; syntenin-dependent exosome formation) [56,57]. Several Rab GTPases are also known to play important roles in intracellular vesicle transport, and thereby can participate in the biogenesis of exosomes through shuttling of endosomes or Golgi vesicles to maturing MVBs [58]. Rab7 specifically has been shown to be involved in these mechanisms [59,60], whereas Rab5, Rab7, Rab11, Rab35 and especially Rab27 have all been found to be involved in vesicular release from donor cells of various types [61,62,63,64,65,66,67,68,69]. However, ESCRT-independent mechanisms of exosomal biogenesis have been both far less characterized and considered less frequently, therefore the majority of exosome studies to date first focus on the ESCRT pathways for mechanistic studies.

The selective incorporation of components into exosomes allows for unique exosomal profiles amongst cell types and can reflect differences in donor cell states, particularly during infection [70,71,72]. Not surprisingly, several viruses have developed mechanisms to hijack exosomal biogenesis and ESCRT pathways/protein components to promote the viral life cycle and budding [73,74,75]. For example, the human immunodeficiency virus (HIV) Gag and the EBOV VP40 proteins have both been found to interact with TSG101, a component of the ESCRT-I complex, and Alix, an accessory ESCRT pathway protein, to facilitate viral release from infected cells [76,77,78,79,80,81,82,83,84,85]. Likewise, numerous viral components from a multitude of infections have been discovered within exosomes and EVs, and have been shown to impact recipient cells and ultimately play a role in the course of pathogenesis [70,71,72]. For this reason, there has been greater focus on the role of EVs during viral pathogenesis, which now represents a rapidly growing field of study for an expanding number of viruses and infectious diseases.

**Figure 1:**
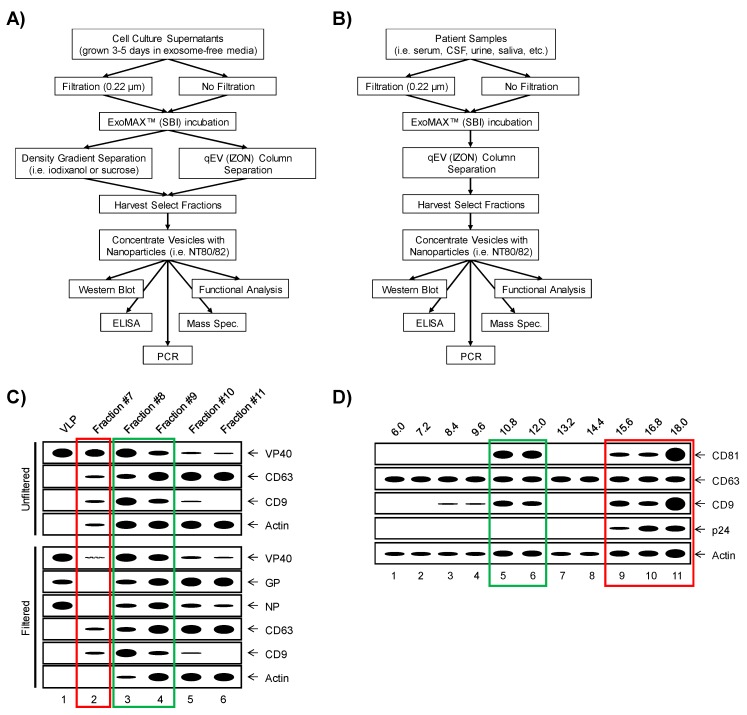
Separation of extracellular vesicles (EVs) from virus for downstream analysis. An optimized a workflow for the separation of EVs away from virus from a variety of backgrounds, including from cell culture supernatants (**A**) and patient samples (**B**). Expected profiles of Ebola virus (EBOV) vs. exosomal proteins when extracellular components (i.e., EV-containing cell culture supernatants or biofluids) are separated by size with qEV (IZON) columns, as outlined in the panel A workflow (**C**). Positive control virus-like particle (VLP) (containing VP40, nucleoprotein (NP) and GP) profiles are shown in lane 1, whereas a typical profile of EV-containing fractions of supernatants that were either unfiltered (top panel) or filtered through 0.22 µm (bottom panel) from cells expressing VP40, NP and GP, followed by separation on qEV size exclusion columns are shown. Expected profiles of mature human immunodeficiency virus (HIV) virions (as indicated by p24 capsid protein) vs. exosomal marker proteins when cell culture supernatants are separated by density, such as by iodixanol or sucrose gradients, as outlined in the panel A workflow (**D**). Profiles demonstrated here are the expected results for iodixanol fractions in increments of 1.2% from 6.0%–18.0%. Fractions where either virions or VLPs are known to elute or sediment are indicated by red boxes. Fractions containing only exosomes and no viral particles are enclosed by green boxes.

The heterogeneity of EVs has forced the development of several purification techniques geared towards the isolation of distinct EV populations. The classic gold standard of EV isolation involves a series of stepwise ultracentrifugation steps to sediment total EV populations, followed by density gradient separation (i.e., iodixanol or sucrose) to further divide EVs into distinct density fractions [86,87]. Using this standardized protocol, separation of EVs from protein or protein-RNA aggregates and potentially viral particles is made possible [87,88,89]. However, this method requires somewhat large sample volumes and therefore may have restricted utility due to the volume limitations associated with in vivo studies. More recently, this protocol has been modified to implement various EV precipitation reagents such as ExoMAX (SBI) to overcome large sample volume limitations [90]. The study of EVs in the context of viral infection requires precise isolation techniques in order to effectively remove any contaminating virions or virus-like particles (VLPs) from the isolated EVs preps. The methods required for virion removal are largely dictated by the overall size of the virion in question and will likely vary between viruses of interest. For example, the Ebola virion is approximately 1–2 µm in diameter, allowing for the vast majority of it to be removed from the supernatant using 1–2 passes through a 0.22 µm filter [91,92,93,94,95,96]. On the other hand, the HIV virion measures only 100 nm in diameter, a size similar to that of exosomes, thereby requiring exclusion through density gradient separation [90,97]. Additionally, investigations into EVs which originate from BSL-4 level pathogens, such as EBOV, have led to the implementation of methods which utilize disposable size-exclusion columns, such as the qEV columns (IZON), which can be used with either samples concentrated by ExoMAX or on raw patient samples to isolate vesicles [96]. The protocols designed to efficiently separate various viruses away from EVs along with expected results are shown in Figure 1.

Utilization of these techniques which allow for separation of EVs away from virus or VLPs has allowed for significant research into the mechanisms by which EVs can promote viral infections. Our lab has recently found that EBOV proteins such as VP40, GP and NP are packaged into EVs released from EBOV-infected cells [95,96,98]. Furthermore, EBOV VP40-associated EVs can elicit bystander lymphocyte apoptosis in recipient, uninfected T-cells and monocytes, thereby potentially representing a novel mechanism contributing to the overall immune deregulation observed in EBOV-infected individuals [95,96,98]. In this manuscript, we review the current findings on EBOV-related EVs, potential contributions of EVs to EBOV pathogenesis and discuss important avenues of future study.

## 2. EVs and VP40

The EBOV VP40 protein functions mainly as the major matrix protein of the virus and is responsible for driving the budding and egress of new viral particles. Expression of VP40 within cells is also known to result in the formation of VLPs, which is amplified by the co-expression of other viral proteins NP and/or GP, and appear virtually indistinguishable from infectious virions [91,99,100,101,102,103,104]. Interestingly, recent studies have shown additional, non-structural functions of VP40 that may play potentially important roles in EBOV pathogenesis. Once such additional role has been shown through the presence of EBOV VP40 within EVs [95,96]. Analysis into the mechanisms behind the packaging of VP40 into EVs, and exosomes in particular, has elucidated numerous interactions of VP40 with host proteins and pathways including exosomal packaging, cell cycle and transcriptional regulation. The outcomes of these interactions have been shown to impact the host cell in a variety of ways, which ultimately have been hypothesized to aid the virus both in replication and spread during infection [95,96]. The connections of VP40 with the host cell to affect its packaging into EVs are summarized here.

### 2.1. VP40 and the ESCRT Pathway

The mechanism of VP40 packaging into exosomes has previously been shown to be ESCRT-driven [95,96]. This is perhaps not surprising, as it has previously been revealed that interactions between the EBOV VP40 late domain and components of the ESCRT pathway such as TSG101, Alix and VPS4 takes place to aid in budding of virions and VLPs. TSG101, part of the ESCRT-1 complex, has been demonstrated to aid in the budding of EBOV virions and VLPs through connections with the P(T/S)AP domain of VP40 [99]. This is very similar to the actions of TSG101 with matrix or associated proteins of other viruses, such as HIV Gag, the open reading frame 3 protein of hepatitis E virus, and the C protein of Nipah virus [78,79,105,106,107]. Alix is also known to arbitrate various cellular and viral protein interactions with the ESCRT machinery for both integration into ILVs and viral budding [108,109]. Indeed, the EBOV VP40 YPx_(n)_L/I late domain motif has been confirmed to recruit Alix to aid in viral budding and egress independently of TSG101 [76]. Furthermore, the vacuolar protein sorting 4 (VPS4) protein (responsible for the final stage of ILV formation and various membrane scission events such as cytokinesis) has been previously implicated to play a role in the budding of EBOV VLPs, as demonstrated by the inhibition of efficient VLP release with the use of a VPS4 dominant-negative mutant [77,83]. In accordance with these previous works, we have demonstrated that VP40 expression leads to changes in TSG101, Alix and VPS4 protein levels and forms. These intracellular changes correlated with expression differences of other exosome and EV-related proteins such as CD63 tetraspanin and Alix in the secreted EVs. Furthermore, overall changes in other ESCRT components (i.e., EAP20 and EAP45 (ESCRT-II) and CHMP-6 (ESCRT-III)) have been observed in cells expressing VP40 [95,96]. Together, these documented ESCRT protein changes were linked to the packaging of VP40 into exosomes and EVs, implicating an ESCRT dependence for this occurrence.

Similarly, although not directly an ESCRT-related protein, efficient release of VLPs has been shown to be aided by the activity of the E3 ubiquitin ligase NEDD4 on the PPxY domain of VP40. This mechanism was shown to take place either at the exclusion of, or was enhanced by, the coordinated activity of TSG101 [99,110]. In further support of this, interferon-stimulated gene 15 (ISG15; a ubiquitin-like protein known mainly for its role in ISGylation) has been shown to inhibit the NEDD4-mediated ubiquitination of VP40 and enhancement of VLP egress [111]. Additional E3 ubiquitin ligases that have been shown to interact with VP40 include Itchy E3 ubiquitin ligase (ITCH) [112], WW domain-containing E3 ubiquitin protein ligase 1 (WWP1) [113] and suppressor of cytokine signaling 3 (SOCS3) [114]. As many proteins in the ESCRT pathway, including those in the ESCRT-0, -I, and -II complexes, are known to detect ubiquitinated, membrane-associated molecules as cargo for packaging into MVBs [51], it stands to reason that VP40, once ubiquitinated by NEDD4 or other host proteins, may serve as a natural target for integration into exosomes by the ESCRT pathway. As of yet, it is unknown if VP40 requires ubiquitination to become packaged into exosomes; however, should ubiquitin prove necessary for this mechanism, it may be interesting to determine if ISG15 or ubiquitin ligase inhibitors are able to prevent the packaging of VP40 into exosomes and downstream effects. Regardless, these data demonstrate that VP40 can utilize many of the same cellular factors to exit the cell through both direct budding from the plasma membrane and through release in vesicles.

### 2.2. VP40 Influence on the Host Cell Cycle

The host cell cycle has often been implicated in viral infections. Indeed, over the years numerous viruses have been shown to dysregulate the cell cycle of host cells in order to help in viral replication, spread, and/or latency. Examples of such viruses include HIV, influenza viruses, human T-cell leukemia virus type 1 (HTLV-1), herpesviruses, human papillomaviruses, hepatitis B and C viruses, infectious bronchitis virus and Adenoviruses [115,116,117,118]. Along these lines, we have also recently shown that cell cycle is altered in cells constitutively producing EBOV VP40. Specifically, VP40 production was shown to ultimately result in a promotion of cell cycle advancement and an overall increased growth rate in host cells [96]. This promotion of cell cycling was largely attributed to an increase in the amounts of cyclin D1 protein, which was directly correlated to the amounts of VP40 being produced within the cell. Levels of cyclin D proteins have been shown to be specifically manipulated by multiple viruses (such as HTLV-1, human neurotropic JC virus (JCV), human respiratory syncytial virus (HRSV), human papillomavirus, and Epstein-Barr virus) to aid in their life cycles [119,120,121,122,123,124]. Additionally, increased cyclin D1 levels have been linked in many cases to oncogenic phenotypes and accelerated cell cycling [125,126,127]. The normal role of cyclin D1 during cell cycle is to bind to its appropriate cyclin-dependent kinase (cdk) partner, cdk2 or cdk4, to activate its kinase activity, and to phosphorylate Rb to stimulate G1 to S phase cell cycle progression [125]. Therefore, upregulation of cyclin D1 as seen in VP40-producing cells is expected to result in increased growth, consistent with observations of these cells. Upregulation of other cyclins in addition to D1, including cyclins E, A, and B1, was also observed in cells producing VP40 [96]; however, attention was focused on cyclin D1 as the D family of cyclins are the first to be upregulated during the initiation of cell cycle from G0.

Consequently, the fact that cyclin D1 is upregulated in cells producing VP40 led to a new set of questions regarding the mechanism of upregulation. Several previous studies have shown EBOV VP40 present within the nucleus of infected cells transiently at early phases of infection [128,129,130]. These works led to closer investigations of nuclear VP40’s presence and role, particularly in relation to cyclin D1 upregulation. It was found that in stably transfected 293T cells, VP40 protein was indeed present in the nucleus at fairly high levels [96]. Furthermore, nuclear VP40 was found to be bound frequently to the cyclin D1 promoter, along with p300 and RNA Pol II at slightly elevated levels. Together, these studies potentially implicate VP40 as a recruiter of transcription factors or activator of transcription of various host genes that may aid in cellular replication and growth [96]. It would stand to reason that this would be beneficial for the virus, as it has been demonstrated that EBOV replicates better in actively dividing cells, whereas inhibition of cell cycle reduced the numbers of progeny virus [131]. Therefore, utilization of VP40 to induce accelerated growth through transcriptional upregulation of cyclin D, such as in epithelial cell types that continue to grow and divide, could represent a previously unappreciated mechanism of pathogenesis for EBOV.

The ESCRT pathway’s role in the release of VP40 exosomes was shown to be mediated in a cell cycle-dependent manner. Levels of ESCRT complex and exosomal marker proteins have been shown to be altered at different phases of the cell cycle, with lower levels present in quiescent or G0 cells compared to those in G1/S or G2/M [96]. Accordingly, lower levels of these proteins were correlated with significantly decreased numbers of EVs at G0 in cells [96]. These trends were true for both normal 293T cells and those constitutively expressing EBOV VP40, although cells expressing VP40 tended to produce vesicles that were both larger in diameter (on average, as determined by the mode of the population by ZetaView NTA analysis) and fewer in number as compared to 293T [96]. Therefore, the most EVs were produced from all cell types during G1/S and G2/M phases of the cell cycle, suggesting that progression through cell cycle may enhance exosome and EV generation. Since VP40 increases the rate of cell cycling, these results could also imply that VP40 can amplify the rate of EV biogenesis by the same mechanism. The augmentation of VP40-containing EV release through cell cycle progression by VP40 could have significant implications for recipient cells, and the immune status of the host in general.

### 2.3. EV-Associated VP40 and Recipient Bystander Cell Effects

As indicated above, during EBOV pathogenesis, a massive bystander lymphocyte apoptosis takes place which is particularly pronounced in fatal cases of EVD. Several potential mechanisms for this have been proposed, including Fas/FasL and TNF-TRAIL interactions, impaired DC/T-cell interactions, and nitric oxide (NO) or viral glycoprotein-induced apoptosis [15,132,133,134]. Recently, VP40-containing EVs have been proposed as an additional possible mechanism for the induction of bystander lymphocyte apoptosis during EVD. This was evidenced by the induction of cell death of both T-cells and monocytes upon incubation with VP40 EVs [95,96,98]. This induction of cell death was shown to be through apoptosis, and the effect was shown to be cell type specific, as recipient 293T cells did not react in the same manner. Indeed, 293T cells had improved viability when treated with VP40 EVs [96]. This stark contrast has interesting implications for how EVs from infected cells may impact pathogenesis within individuals. There is the potential for EVs containing VP40 from EBOV-infected cells to travel to recipient immune cells and thereby reduce the pool of surveilling T-cells and monocytes, allowing for the virus to replicate unhindered. Should this mechanism of immune modulation by EVs from infected cells prove true, it may be even more relevant in cases of recurrent or persistent infections. Hypothetically, if VP40-containing EVs from infected cells are able to increase the viability of recipient epithelial cells, this may allow these cells to be more permissive to long-term infection. Contrastingly, the same VP40 EVs may be able to suppress the host immune surveillance, thereby allowing any persistently infected cells to remain and hide, especially within immune privileged sites. This potential model supporting continued infections has been described at length elsewhere [98].

## 3. EVs and NP

Recently, we have demonstrated the presence of EBOV NP in EVs from EBOV-infected cells [96]. The NP of EBOV functions in the assembly of the viral nucleocapsid along with VP30, VP24 and VP35 [135]. Expression of EBOV NP on its own elicits the formation of nucleocapsid-like structures, but co-expression of VP35 and VP24 elicits the formation of nucleocapsids that are more similar to those found in actual virus particles [136,137]. As soon as the viral genome is synthesized in cytoplasmic inclusion bodies, NP encapsidates it with strong affinity which is regulated by a system of phosphorylation and de-phosphorylation [135]. Interestingly, EBOV NP binds RNA in the absence of the other proteins [138]. Each NP monomer covers a six-base RNA fragment and holds the genome in an ordered, left-handed helical shape [139]. The structure of NP has been studied in depth to reveal specific functions of its N- and C-terminal domains. The N-terminal domain is highly conserved and shares structural homology with other *Mononegavirales* viruses. It contains two lobes heavy with α-helices, which drive the binding and encapsidation of RNA. There is also a hydrophobic coiled-coil motif at the N-terminus, which may be a binding site for VP35 to inhibit NP-RNA interactions during transcription and replication [136,140,141]. The NP C-terminus is poorly conserved and shares no homology with other viruses of the same order. Alternating α-helices and β-sheets allow this domain to act as a center for protein-protein interactions, particularly with VP35 or VP40 during nucleocapsid assembly and packaging into virions [140,142,143]. While the role of EBOV NP as an RNA-binding protein has been well characterized, we speculate that there may be additional roles for EBOV NP that have yet to be uncovered and may be consequential for cells that may incorporate this protein into EVs.

The nucleoproteins from Crimean-Congo hemorrhagic fever virus (CCHFV) and Lassa virus (LASV), two unrelated hemorrhagic fever viruses with high mortality rates, were recently described to have nuclease functions in addition to their roles in nucleocapsid formation [144,145,146]. Structural analyses of these two nucleoproteins revealed similarities to known nucleases and subsequent studies revealed both NPs to have nuclease functions. In the case of LASV NP, its dsRNA-specific exonuclease function was important for blocking the activation of type I IFN responses [145]. Previous structural studies of EBOV NP have focused on an N-terminal “core” domain [141] or C-terminal tail [142], leaving the region comprised of amino acids 405–641 relatively uncharacterized [141]. It would be interesting to analyze the structure of this region to see if additional functions for EBOV NP can be imputed based on similarity with other protein domains. Regardless of the lack of a structure for this region, studies have shown that EBOV NP mutants that lack amino acids 451–600 or 451–739 inhibit minigenome-driven reporter gene expression in a dose-dependent manner [136]. EBOV NP lacking amino acids 451–600 or 506–739 failed to form properly-sized nucleocapsids [136,137], which taken together indicates an important role for this region in the function of NP and nucleocapsid formation, but a specific role has yet to be revealed. 

Several lysine residues in EBOV NP were recently described to be acetylated by recombinant histone acetyltransferases (HAT) in vitro. K513 and K617 were found to be acetylated by P300/CREB-binding protein (P300/CBP) and/or P300/CBP-associated factor (PCAF) [147]. These two acetylated lysine residues reside within a region that is less conserved among members of the Ebola virus genus, although K513 is conserved between EBOV and Bundibugyo virus (BDBV) but not Sudan virus (SUDV) (USAMRIID; BLASTp unpublished observation). Furthermore, as discussed above, K513 and K617 fall in a region of EBOV NP that is not important for its RNA-binding function or association with other EBOV proteins. Regardless, acetylated lysine residues can function as binding sites for bromodomain-containing proteins and as such these residues in EBOV NP may be docking sites for other host proteins. Many bromodomain-containing proteins have chromatin remodeling and/or transcription modification functions [148] and one such protein, Brd3, has a critical function in the elicitation of type I IFN production [149]. Infection of macrophages with Vesicular stomatitis virus (VSV) downregulated Brd3 expression, which in turn inhibited the ability of macrophages to produce IFNβ [149], demonstrating that Brd3 can be targeted in viral infections to limit type I IFN responses. It is therefore tempting to speculate that the acetylation of various lysine residues in EBOV NP may act as decoy docking sites for Brd3 to inhibit type I IFN responses. Brd3 is primarily a nuclear protein, but EBOV NP has been demonstrated to traffic to perinuclear areas in infected cells [129] which may give EBOV NP access to Brd3 prior to its nuclear translocation. A cellular interactome analysis of EBOV NP with cellular proteins identified SMYD3 [150], a histone methyltransferase, which is reported to recruit Brd4 to positively regulate transcription of target genes [151]. This interactome study sets a precedent for the interaction of EBOV NP with proteins involved in transcription regulation but nonetheless also establishes a potentially interesting pattern of NP involvement with hitherto unheralded cellular processes. 

While no direct IFN-antagonist function has been described based on structural studies of EBOV NP, EBOV NP has been implicated in IFN antagonism based on mutations that were acquired upon the adaptation of wild-type EBOV to a mouse-adapted variant. Mouse-adapted EBOV has mutations in NP and VP24 that render the virus lethal in wild type mice [152]. A recombinant virus bearing only the NP mutation, denoted “WT-NP_MA_,” found in mouse-adapted EBOV, demonstrates resistance to type I IFN when grown in murine macrophages treated with type I IFN [152]. WT-NP_MA_ virus did not grow as well when IFN was introduced prior to or after infection, indicating that if the IFN response is activated in advance, any IFN antagonism is less effective. The WT-NP_MA_ mutation in mouse-adapted EBOV changed a serine to glycine, so why this mutation is so consequential is not understood since lysine acetylation would not have been changed. Nevertheless, this demonstrates an involvement of EBOV NP with type I IFNs.

Though any direct IFN-antagonistic function of EBOV NP has yet to be described, the release of EVs containing EBOV NP from EBOV-infected cells presents a tantalizing possibility that such EVs may carry a payload that can negatively regulate dose-dependent levels of type I IFN responses in recipient cells. The number of NP molecules or indeed their monomeric or multimeric state in EVs has yet to be characterized, so it is unclear how effective such immunoregulation in uninfected recipient cells based on EBOV NP in EVs might be. Given that various EBOV proteins have diverse roles in immune evasion and suppression [135], any change in “type I IFN competency” in cells neighboring an EBOV-infected cell may predispose them to infection in turn. EBOV NP in EVs may therefore be an early immunosuppressive strike that helps predispose the infected host to develop a fulminant infection.

Alternatively, the role of any RNAs bound by EV-associated NP have yet to be elucidated, but if present may possibly affect recipient cells. As mentioned above, NP strongly associates with viral RNA as it is replicated in inclusion bodies within the cytoplasm. However, this affinity of RNA by NP has been shown to not be specific for viral RNA only [143,153]. Therefore, it is possible that the NP packaged into EVs may be bound to a multitude of host RNAs of various sizes and with functional characteristics. In the future, sequencing of the RNAs bound by EV-associated NP would be particularly interesting to determine any potential effects these RNAs may have in recipient cells.

## 4. EVs and GP

As was recently shown with NP, we have also demonstrated EBOV GP within EVs from EBOV-infected cells [96]. Entry of filoviruses into cells is dependent upon the GP protein displayed on the viral surface. GP is translated from a single open reading frame as a GP_0_ precursor. GP_0_ is then processed through the endoplasmic reticulum (ER) and Golgi apparatus, where it is cleaved by furin-like proteases to produce two subunits connected by a disulfide bond: GP_1_ and GP_2_ [135]. GP_1_ is an ectodomain responsible for interactions with cell receptors, while GP_2_ is the transmembrane domain and important for membrane fusion [9,154]. A trimer of GP_1,2_ molecules compose the mature GP protein. The N-terminal section of the GP_1_ subunit, also known as the core protein, interacts with GP_2_ and forms the head of the protein that interacts with receptors. The C-terminus of GP_1_ is known as the mucin domain, is positioned on top of the GP_2_ head, and is heavily glycosylated, thus producing a ‘glycan cap’ [154,155,156]. GP can be processed differentially, however. As mentioned above, only ~20%–25% of GP products are the membrane-anchored GP_1,2_ form, whereas the rest of GP proteins are in dimeric secreted forms, with ~70% sGP and ~5% ssGP [7,8,9,10]. Furthermore, a shed form of GP_1,2_ (shed GP) is generated by a cleavage event at the cell membrane by host tumor necrosis factor-a converting enzyme (TACE). All four of these GP forms contain the same first 295 residues of the N-terminus, although their C-termini differ, resulting in different functions. Only GP_1,2_ and shed GP possess the heavily glycosylated mucin-like domains which mask the GP epitopes [10].

Filoviral entry is accomplished through several steps: attachment, uptake and fusion. First, target cell attachment is mediated by numerous possible nonspecific attachments between the GP_1_ glycan cap and host cell surface proteins including primarily C-type lectin family members (i.e., hMGL, asialoglycoprotein, DC-SIGN, DC-SIGN(R), L-SIGN and L-selectin), but also may involve various β1-integrins, Axl and TIM-1 and -4. These later proposed interactions may be even more nonspecific and could involve interactions with phosphatidyl-serine on the filoviral membrane [154,155]. Uptake of EBOV is accomplished through a micropinocytosis-like mechanism, characterized by actin-dependent plasma membrane ruffling and blebbing to engulf bulk cargo and extracellular fluid [157,158]. Initiation of this uptake has been shown to be strictly GP-dependent, but the specific mechanism is not well characterized [155,158]. The virus is then trafficked to late endosomes and/or lysosomes, as indicated by co-localization first with Rab5-positive early endosomes and then Rab7/LAMP-1-positive late endosomes [157]. Within endosomal vesicles of lowered pH and reducing conditions, proteolytic cleavage of GP_1,2_ is performed by host cysteine proteases cathepsin B and/or cathepsin L [154]. This cleavage event removes portions of the glycan cap and mucin-like domain of GP_1_ [154]. Once the head portion of GP_1_ has been exposed, viral attachment to the Niemann-Pick C1 (NPC1) host cell late endosomal/lysosomal membrane protein can take place. Due to the proposed specificity and importance of NPC1 for filoviral entry, this receptor is a promising target for antiviral development [154,155,159]. Following binding to NPC1, viral fusion and release of the nucleocapsid into the cytoplasm is initiated by conformational changes in the GP_2_ subunit and fusion with the endosomal membrane [154,155,160].

**Figure 2:**
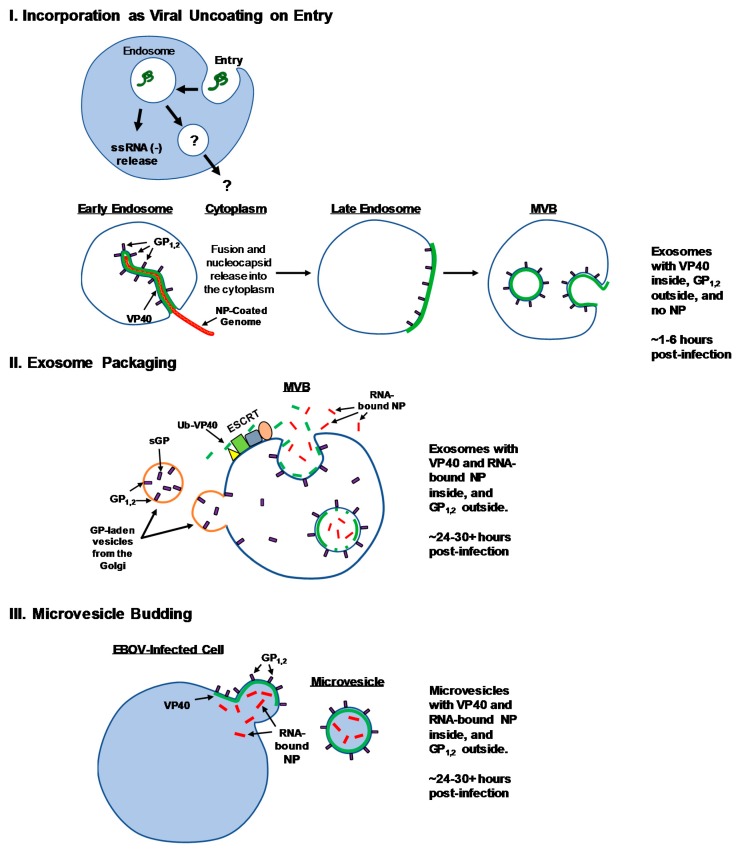
Possible mechanisms of unconventional EBOV protein secretion. Three proposed mechanisms of packaging of EBOV proteins VP40, GP, and NP into EVs are illustrated along with hypothesized times of release. (**I**) Incorporation as viral uncoated on entry: Entry of infectious virions or VLPs into host cells through endosomes results in cleavage of the outer portions of GP_1,2_, binding to the host receptor, and fusion with the endosomal membrane to release the viral nucleocapsid into the cytoplasm. Viral GP and VP40 proteins remaining within the endosome after nucleocapsid release may become packaged into exosomes as a result of inward budding of the endosomal membrane, resulting in extracellular release an estimated 1–6 h post-infection. (**II**) Exosome packaging: classical loading of cargo into exosomes through endosomal sorting complexes required for transport (ESCRT) pathway (potentially ubiquitinated VP40; Ub-VP40), Golgi vesicle transport (GP_1,2_ and soluble GP (sGP)), and other mechanisms (NP). Resulting hypothesized exosomes would be released approximately 24–30+ h post-infection (after viral translation of new protein components), and contain GP_1,2_ on the surface, and VP40 and NP on the inside. The secretion of free sGP may also take place through fusion of multivesicular bodies (MVBs) with the plasma membrane. (**III**) Microvesicle budding: The outward blebbing of microvesicles from the plasma membrane containing nontraditionally assembled EBOV components. GP_1,2_ is estimated to be oriented on the surface, with VP40 and NP on the inside. Microvesicles are estimated to be released approximately 24–30+ h post-infection. Additional details of these models are discussed in the text.

While we have shown the presence of GP within EVs, we do not yet know the form(s) of GP that become packaged or the mechanism(s) of their packaging. GP is synthesized in the ER, followed by modification in the Golgi apparatus, where it is then transported in vesicles to the plasma membrane [154,161,162]. However, vesicles from the Golgi do not necessarily target only to the cell surface. Alternative targets for Golgi vesicles include early and late endosomes, MVBs, and lysosomes [163,164]. Indeed, GP translocation to endosomes as a consequence of vesicular trafficking from the Golgi has been postulated previously [160]. Furthermore, after post-translational processing, Golgi vesicles are known to contain not only fully formed GP_1,2_, but also sGP [162,165]. As late endosomes can go through inward budding processes mediated by ESCRT complexes, it stands to reason that GP-laden vesicles from the Golgi bound for late endosomes can thus become MVBs, and in turn exosomes that are released from infected cells. Alternatively, GP targeted to the cell surface can likely take part in microvesicle biogenesis by outward budding of the plasma membrane in addition to its normal role in VLP or virion budding. It has been previously shown that full-length GP accumulates in the ER 24 h after transfection, but was not detected in the Golgi or endosomes at this time point [166]. Likewise, the emergence of new viral particles after infection has been observed at 48 h [129]. Therefore, GP is likely processed through the Golgi apparatus and trafficked to various cellular locations sometime between these time points. It is tempting to speculate that GP, and potentially other viral proteins including VP40 and NP, may be packaged into exosomes and other EVs and subsequently released before the assembly and egress of infectious viral particles. The proposed mechanisms and hypothesized timing of GP incorporation into EVs are included in Figure 2.

Beyond the potential timing and mechanisms of integration of GP into EVs, there are significant functional possibilities for GP-studded EVs during pathogenesis. It has been observed by several groups that GP, either in membrane-bound GP_1,2_ or shed GP forms, is able to induce substantial immune activation of monocytes, macrophages, and DCs, resulting in chemokine and cytokine secretion through TLR4 stimulation [167,168,169]. Treatment with sGP did not induce the same phenotype, and it was determined that the glycosylation of the mucin-like domain was essential for this to occur [167]. GP-TLR4 induction of cytokines and chemokines functioned to recruit CD11b^+^ macrophages and CD11c^+^ DCs in vivo [168]. Moreover, GP-TLR4-stimulated cells were shown to potentially have enhanced budding of VLPs [169]. Along these lines, a recent study demonstrated that shed GP-treated monocytes were triggered into differentiation through TLR4 activation [170]. Interestingly, previous work has shown that monocytes are somewhat inefficiently infected, whereas macrophages and DCs are much more permissive to productive entry and replication of EBOV, and that uptake of EBOV by monocytes stimulates their differentiation. Maturation of these cells in turn upregulates both the host cell receptor NPC1 and cathepsin B, which presumably aids in viral entry, fusion, and release into the cytoplasm from the endosome [171]. It is therefore not surprising that shed GP-TLR4 induction of uninfected monocyte differentiation also enhanced new EBOV infection and eventual cell death [170]. Together, these studies imply that stimulation of the TLR4 pathway by GP_1,2_ or shed GP in myeloid cells potentially serves to not only prime them for new infection and differentiation, but also to promote cytokine release to recruit additional immune targets for the virus. Additional roles for GP in pathogenesis have also been described. It was found that shed GP was able to increase the vascular permeability of endothelial cells both directly and indirectly through cytokines (both pro- and anti-inflammatory) released from myeloid cells agitated by TLR4 binding [167]. Furthermore, glycosylated forms of GP have been shown to play roles in both the inhibition of natural killer (NK) cells [172] and potentially in inducing T-cell death through TLR4 binding [173]. Should glycosylated forms of GP be present on EVs, it is possible that similar mechanisms to these could be at work. Lastly, secreted forms of GP have been widely speculated to play roles in immune evasion mainly through acting as decoys for antibodies during the course of infection [174,175]. If GP proves to be exposed on the surface of EVs, with either one or multiple epitopes present, it is logical to assume that these could also serve as decoys for the virus to aid in immune evasion.

## 5. Remaining Questions

While the presence of EBOV VP40, NP, and GP within exosomes and/or EVs has been demonstrated, many questions still remain about the mechanisms of packaging and functional consequences of these vesicles. Firstly, what are the forms of these viral proteins that are primarily found encapsulated within or exposed upon the surface of EVs? Is there one main form or multiple for each of these proteins? Are they found as monomers or as multimeric units, particularly in the case of VP40 and NP? What are the post-translational modifications found on these proteins, and what are the effects enacted by those modifications? The surface has hardly been scratched on the numerous possibilities and implications that exist. It is likely that in-depth proteomics studies will be necessary to fully appreciate the breadth utility of the forms of EV-associated EBOV proteins.

Another important consideration is the additional cargo within EVs that has not yet been characterized. While VP40, NP, and GP have been found, what additional cargo may be specifically packaged within EVs as a result of these viral proteins? It has not yet been determined whether EBOV packages any of its four other proteins (VP24, VP30, VP35 and L) into EVs. VP30 and L are important for the translation of viral products, whereas VP24 and VP35 have significant pathogenic functions in IFN antagonism and impairment of signaling. Due to its size, it seems unlikely that L will be found within EVs of <220 nm diameter. However, should VP24 or VP35 be found within EVs, these could impart substantial immune interference within recipient cells, particularly if they are potential host cells for the virus.

Outside of cargo from viral origin, host cell cargo is another important consideration. Previous work has observed an upregulation of several cytokines within EVs from VP40-producing cells [96]. Among the upregulated cytokines were TGF-β1, IL-15, MCP-1, and IFN-γ—cytokines known to be upregulated during EVD, particularly in fatal cases [4,14,176,177,178]. IFN-γ is a proinflammatory cytokine that induces both MHC class I, class II, and IL-12 upregulation in activated macrophages, as well as triggers IL-12R expression on T-cells [179,180]. It was classically thought that mainly T-cells (Th1) and NK cells were responsible for the production of IFN-γ to support the function of CD4 and CD8 T-cells; however, many other cell types including smooth muscle cells, endothelial cells, and renal cells have been shown to make type II IFN [181,182,183,184,185]. The importance of IFN-γ for bridging the innate and adaptive immune responses is well-known, but in EVD, IFN-γ has been measured to be elevated in fatal cases, with loss of T-cell populations shortly after systemic detection of the cytokine [176,186]. IFN-γ is also pro-apoptotic, and has been proposed to potentially promote bystander T-cell death in EBOV infection [176]. IL-15 induces proliferation and differentiation of B-cells, T-cells and NK cells, while also boosting CD8 cytolytic and memory T-cell activity [187]. MCP-1’s main role is as a chemoattractant, responsible for inducing the migration and infiltration of monocytes/macrophages [188]. Both IL-15 and MCP-1 have been found to be hyper-secreted in fatal EVD [15,132]. Finally, TGF-β1 participates in anti-inflammatory immune regulation through differentiation of Treg subsets, but also has pro-inflammatory action through stimulation of Th17 cell development [176]. While TGF-β1 has been shown to be enhanced in EBOV-infected hepatocytes, the functional role of this cytokine in EVD pathogenesis is not known [189]. As the initial experiments finding these cytokines associated with EVs were not in a myeloid cell background, further investigation into the possible cytokines upregulated in infected or transfected myeloids should yield interesting results. The finding of these cytokines within EVs from EBOV protein-producing cells is not unique, as many others have observed similar results in EVs and exosomes from a multitude of cell types and disease backgrounds [190,191,192,193,194]. A recent study by Fitzgerald et al. [195] characterized the presence of 33 cytokines on the surface vs. encapsulated within EVs vs. secreted as soluble forms from in vitro (T-cells and monocytes), ex vivo (tissue explants from amniotic, placenta, tonsillar and cervical origins), and in vivo (plasma and amniotic fluid) samples. Their results suggested that, depending upon the system in question and the stimulus present, various cytokines could be released either as soluble or EV-associated forms. Additionally, EV-associated forms displayed greater stability over time, and were bio-active in recipient cells [195]. Interestingly, Fitzgerald et al. [168] showed that monocytes stimulated with LPS shifted to release MCP-1 from a soluble form to more EV surface-associated forms, along with increased overall levels of EV secretion. This observation is nicely in line with previous literature that demonstrated the increased release of MCP-1 and other chemokines/cytokines in shed GP-treated mice. Since it is known that shed GP can bind to and activate TLR4 similar to LPS [167,168,169], these collective results may imply that shed GP, virions, and/or GP-associated EVs may be able to bind to TLR4 in vivo, thus activating monocytes to release higher levels of MCP-1 on EV surfaces in order to recruit additional monocytes to the local site of infection, thereby drawing future targets to the virus. In the future, determination of the levels and potential role of EV-associated cytokines during EBOV pathogenesis may be of great interest, as it is likely that the encapsulation of cytokines in EVs will prolong their stability to allow for activation of distant recipient cells in various physiological compartments.

Further questions into the type of vesicle primarily utilized for EBOV protein secretion are also warranted. Initial experiments have focused on the role of exosomes due to the more developed literature on characteristics and biogenesis of these vesicles; however, other important types of vesicles such as microvesicles or secretory autophagosomes may be equally or of greater importance to the pathogenesis and unconventional secretion of EBOV components. We consider three likely scenarios for the incorporation of EBOV VP40, NP and GP into EVs for secretion. (1) The incorporation of viral products as a side-effect of viral uncoating in the early endosome upon entry into host cells. After macropinocytosis, host cathepsins cleave the outer portion of the GP_1_ mucin-like domain to reveal the receptor binding site, which then binds to host receptor NPC1, allowing fusion of the viral envelope with the endosomal membrane and release of the nucleocapsid into the cytoplasm. Logically, VP40 and GP of the virion will still remain within the endosome after release of the genome into the cytoplasm. Should this endosome undergo inward budding afterwards, these components may then become integrated into nascent ILVs, which may then become exosomes containing cleaved GP_1,2_ on the outside and VP40 on the inside. (2) Classical packaging into exosomes via ESCRT-dependent mechanisms. In this scenario, VP40 (potentially targeted by ubiquitination) becomes packaged into maturing ILVs through the concerted effort of ESCRT complexes, whereas both membrane-bound GP_1,2_ and free sGP may become integrated into MVBs through Golgi vesicles. Cytosolic NP (potentially bound to RNA) may become packaged into exosomes through an as-of-yet uncharacterized mechanism. Final biogenesis of exosomes by this model will result in exosomes that have GP_1,2_ on the outside, and VP40 and RNA-bound NP on the inside. Upon MVB fusion with the plasma membrane, sGP may also be released in a free form. (3) Budding of microvesicles from the plasma membrane. As VP40, GP, and NP are all well-characterized to associate and assemble at the plasma membrane, it stands to reason that should some factor during assembly be defective, or if the host cell directs the budding process, that microvesicles may be released rather than the classical VLP. We hypothesize that these microvesicles will vary widely in diameter, although they will also have the same orientations of packaged EBOV proteins as would be present during classical exosomal biogenesis. As a conceptual distinction, we therefore propose that although these microvesicles may bud from the plasma membrane and contain the same assembly of viral proteins as VLPs, that they are both morphologically and functionally distinct. We would suggest that the difference between VLPs and EBOV microvesicles or other EVs is largely due to the driving force of secretion and final morphology—VLPs are filamentous and budding is driven by VP40, whereas EVs are roughly spherical and budding is driven by the host cell. During infection, one or multiple combinations of these proposed pathways likely takes place, and the extent to which each, if any, contribute to the observed EVs generated in experimental models and in vivo remains to be determined. The three hypothesized mechanisms of unconventional EBOV protein secretion have been summarized in Figure 2.

The type of vesicle(s) utilized by EBOV proteins to be released from infected cells may have significant ramifications not only for the additional cargo involved (of both host cell and virus origin), but also the timing of release in comparison to infectious virus or VLPs. As mentioned above, we propose that viral components may be packaged into and released from infected cells in various EVs at time points earlier than the release of virions. Since virus release has been observed starting at ~48 h [129], it stands to reason that, since the host cell is continually creating EVs, during the generation of viral proteins to be integrated into new virions, many of these components may be packaged into EVs simultaneously. Should some viral components become packaged into exosomes during entry as proposed in mechanism #1 (Figure 2), these especially are likely to be released relatively quickly upon infection, perhaps even before new viral translation begins. If EVs containing GP are released prior to virion release, it is possible that they may be able to then act as a tool to recruit and differentiate/prime future targets of the maturing virions. Alternatively, should EVs containing VP40 be released earlier than new virus, they may be able to either wipe out sentinel immune cells that are susceptible, or accelerate the cell cycling of recipient epithelial cells to act as future sites of infection.

The pathogenic potential of EBOV EVs is not limited to acute disease. As described above, many cases of recurrent, asymptomatic or persistent infections have arisen. The ability of EBOV to remain clinically latent in immune-privileged sites has not yet been well characterized, but a few recent studies have offered possible hints to this effect. One recent study showed that retinal pigment epithelial cells could potentially serve as reservoirs for productive EBOV infection, and that apoptotic signaling cascades were downregulated in these cells. Additionally, infected retinal cells were induced to release several pro-inflammatory cytokines and chemokines [196]. In asymptomatic NHPs, persistent EBOV infection and replication along with systemic inflammation was found in monocyte/macrophage (CD68^+^) cells in multiple compartments including in the vitreous human and adjacent tissue, the epididymis, and the brain [197]. If retinal pigment epithelial cells or adapted CD68^+^ cells resident in immune-privileged sites are able to sustain EBOV infection, perhaps through VP40 upregulation of cell cycle or other transcriptional events that downregulate cell death, these cells could then release a variety of EVs containing multiple EBOV components, which can then either (1) recruit and/or prime neighboring cells for infection through differentiation or other mechanisms; (2) induce apoptosis in surveilling T-cells; (3) continually barrage neighboring or distant sites with inflammatory mediators such as cytokines or chemokines; or (4) act as decoys for any GP-targeting antibodies produced in the convalescent host. Ultimately, any or all of these mechanisms could potentially allow for the lingering presence of EBOV and prevent its complete clearance for prolonged timeframes.

## 6. Conclusions

We have reviewed here the current evidence of EBOV VP40, GP and NP protein packaging into host cell EVs during the course of infection, and their possible functional effects within infected hosts. The capacity of the EBOV protein-containing EVs to exacerbate EBOV pathogenesis may be many-fold in a variety of situations. Depending upon the type of donor cell, stage of infection, physiological site, and recipient cell, EVs from infected cells could potentially aid in the spread of infection through recruitment and priming of new target host cells, downregulation and/or upregulation of systemic inflammation, and by decimation of the immune cell populations in general. These effects could be important not only in acute infection, but also during persistent or asymptomatic infections. For example, during acute infection, infected cell EVs containing EBOV proteins could potentially be responsible for both the killing of bystander T-cells and the priming and recruitment of myeloid cells for subsequent infection, thus allowing the virus to spread unchecked systemically. Alternatively, during persistent infection in immune privileged sites, infected cells may release low, consistent levels of EBOV protein-associated EVs, which can then potentially travel to peripheral areas and simultaneously destroy surveilling immune populations and serve as immune decoys for any circulating antibodies against the viral GP. Both of these possibilities would represent novel mechanisms which could allow for the virus to effectively evade host immune responses to infection.

As this is a relatively new field, there are many diverse avenues for future study remaining. In particular, identification of the full assortment of EBOV proteins and their forms/orientations that may be packaged into EVs, as well as the functional consequences of cells receiving those EVs is of significant interest. Additionally, characterization of the subtype (i.e., exosome vs. microvesicle) and biogenesis pathway of the EVs mainly responsible for aiding in pathogenesis of the virus will become important, particularly if potential therapeutics will be aimed at this newly identified area. While there are a multitude of questions that remain to be addressed, the evidence thus far certainly shows that the effects of EBOV protein-containing EVs are clearly separate from those enacted by VLPs or virions, and therefore these EBOV EVs likely play their own important role during pathogenesis. For these reasons, it will likely become increasingly important for future investigations into the effects of various types of EVs or VLPs to carefully utilize specific isolation procedures so as to not result in ambiguous or misleading findings.
